# Supplementary material for: Root Transcriptional and Metabolic Dynamics Induced by the Plant Growth Promoting Rhizobacterium (PGPR) Bacillus subtilis Mbi600 on Cucumber Plants
Source: Plants (Basel). 2022 Apr 30;11(9):1218. doi: 10.3390/plants11091218 (PMC9102019; doi:10.3390/plants11091218)
Supplement: Supplementary file 1 [file plants-11-01218-s001.zip › Suppl Tables.pdf]

Table S1. List of oligonucleotides designed and used in the study.

Gene ID	Primer sequence (5' – 3')	Encoding protein	Product size
**Csa_5G643380**	GGGCGGAGTCAAGAGAGCGT<br>TGCCACCACATCTGACCTGCT	endo-1,3(4)-beta-glucanase 3	146
**Csa_2G010370**	GCCCTTGAATTGGCGCAAACAC<br>TTCGACCTGATGGGCCGACG	pathogenesis-related protein PR-4	68
**Csa_2G406640**	AGACGCCGAGAAGGACGCTC<br>TCGGCGCAAGAGACTCGACC	peroxidase	117
**Csa_3G743950**	TTAACGAGCGGTCCAGGCCA<br>ACCGCAATCGCCAGTCATGC	thaumatin-like	165
**Csa_2G011420**	AGAGCTTTGCCCTCCGACGA<br>GGCCACCCCACAACTTGTGC	auxin-responsive protein IAA4	119
**Csa_7G007930**	ACAACATCCGCCGCATCGTG<br>CGCGGTCACTCGAGCCTTCT	indole-3-acetic acid-induced protein ARG7	71
**Csa_3G866530**	ATCCCTTCCGACGTACCCGC<br>GACTCGTCGCAAGGGATTGCC	auxin-responsive 6B	176
**Csa_3G035310**	ACCGGGTTCGCTCTACACCG<br>CGCCTGCAGCAATTTCCGGA	auxin-responsive protein SAUR32	143

Table S2. Gene Ontology (GO) enrichment results of up-regulated differentially expressed genes (DEGs) in *Bacillus subtilis* MBI600 (*Bs* MBI600) - treated cucumber roots. DEGs are categorized according to biological process and molecular function.

**Biological process**								**Molecular function**			
**24 hours**				**48 hours**				**24 hours**			
**Enrichment FDR**	**Genes in list**	**Total genes**	**Functional Category**	**Enrichment FDR**	**Genes in list**	**Total genes**	**Functional Category**	**Enrichment FDR**	**Genes in list**	**Total genes**	**Functional Category**
8,90E-03	6	20	Regulation of transmembrane transport	2,20E-05	71	1247	Regulation of macromolecule biosynthetic process	5,20E-04	69	1295	DNA binding
1,60E-02	57	1247	Regulation of macromolecule biosynthetic process	2,20E-05	72	1234	Regulation of nucleobase-containing compound metabolic process	5,20E-04	35	496	Transcription regulator activity
1,60E-02	3	7	Amine transport	2,20E-05	110	2223	Regulation of biological process	6,80E-04	32	451	DNA-binding transcription factor activity
1,60E-02	59	1234	Regulation of nucleobase-containing compound metabolic process	2,20E-05	77	1389	Regulation of nitrogen compound metabolic process	3,10E-03	100	2230	Nucleic acid binding
1,60E-02	58	1268	Regulation of cellular biosynthetic process	2,20E-05	70	1200	Regulation of RNA metabolic process				
1,60E-02	3	7	Regulation of organic acid transport	2,20E-05	69	1179	Regulation of transcription, DNA-templated				

1,60E-02	3	7	Amino acid export across plasma membrane	2,20E-05	69	1186	Regulation of nucleic acid-templated transcription
1,60E-02	5	19	Regulation of ion transmembrane transport	2,20E-05	71	1240	Regulation of cellular macromolecule biosynthetic process
1,60E-02	5	22	Regulation of ion transport	2,20E-05	69	1186	Regulation of RNA biosynthetic process
1,60E-02	4	10	Regulation of anion transport	2,90E-05	77	1407	Regulation of primary metabolic process
1,60E-02	8	62	Negative regulation of transcription, DNA-templated	3,30E-05	71	1268	Regulation of cellular biosynthetic process
1,60E-02	6	37	Regulation of transport	3,30E-05	71	1271	Regulation of biosynthetic process
1,60E-02	57	1200	Regulation of RNA metabolic process	3,70E-05	71	1279	Transcription, DNA-templated
1,60E-02	3	7	Regulation of amine transport	3,70E-05	34	442	Ion transport
1,60E-02	3	7	Regulation of amino acid transport	3,80E-05	72	1312	Regulation of gene expression
1,60E-02	55	1179	Regulation of transcription, DNA-templated	3,80E-05	77	1445	Regulation of cellular metabolic process

1,60E-02	3	7	Regulation of amino acid export	3,80E-05	71	1295	RNA biosynthetic process
1,60E-02	4	14	Erythrose 4-phosphate/phosphoenolpyruvate family amino acid catabolic process	3,80E-05	78	1470	Regulation of macromolecule metabolic process
1,60E-02	8	68	Negative regulation of RNA biosynthetic process	3,80E-05	117	2478	Biological regulation
1,60E-02	56	1186	Regulation of nucleic acid-templated transcription	3,80E-05	71	1291	Nucleic acid-templated transcription
1,60E-02	8	68	Negative regulation of nucleic acid-templated transcription	3,80E-05	26	293	Cation transport
1,60E-02	3	7	Regulation of amino acid transmembrane transport	6,10E-05	19	178	Metal ion transport
1,60E-02	4	9	Regulation of anion transmembrane transport	1,00E-04	77	1488	Nucleobase-containing compound biosynthetic process
1,60E-02	56	1240	Regulation of cellular macromolecule biosynthetic process	1,10E-04	82	1622	Heterocycle biosynthetic process
1,60E-02	56	1186	Regulation of RNA biosynthetic process	1,10E-04	97	2013	Regulation of cellular process
1,60E-02	4	14	L-phenylalanine catabolic process	1,50E-04	84	1689	Organic cyclic compound biosynthetic process

1,60E-02	58	1271	Regulation of biosynthetic process	1,70E-04	78	1543	Regulation of metabolic process
1,60E-02	90	2223	Regulation of biological process	2,80E-04	80	1617	Aromatic compound biosynthetic process
1,70E-02	61	1389	Regulation of nitrogen compound metabolic process	4,60E-03	91	2063	Cellular nitrogen compound biosynthetic process
1,70E-02	8	70	Negative regulation of RNA metabolic process	7,30E-03	19	258	Ion transmembrane transport

Table S3. Gene Ontology (GO) enrichment results of down-regulated differentially expressed genes (DEGs) in *Bacillus subtilis* MBI600 (*Bs* MBI600) - treated cucumber roots. DEGs are categorized according to biological process and molecular function.

**Biological process**							
**24 hours**				**48 hours**			
**Enrichment FDR**	**Genes in list**	**Total genes**	**Functional Category**	**Enrichment FDR**	**Genes in list**	**Total genes**	**Functional Category**
1,20E-07	115	1247	Regulation of macromolecule biosynthetic process	9,30E-06	7	14	Erythrose 4-phosphate/phosphoenolpyruvate family amino acid catabolic process
1,20E-07	114	1234	Regulation of nucleobase-containing compound metabolic process	9,30E-06	7	14	L-phenylalanine catabolic process
1,20E-07	116	1268	Regulation of cellular biosynthetic process	3,30E-05	7	17	Aromatic amino acid family catabolic process
1,20E-07	112	1200	Regulation of RNA metabolic process	9,90E-05	8	30	Alpha-amino acid catabolic process
1,20E-07	111	1179	Regulation of transcription, DNA-templated	9,90E-05	7	21	Erythrose 4-phosphate/phosphoenolpyruvate family amino acid metabolic process
1,20E-07	112	1186	Regulation of nucleic acid-templated transcription	9,90E-05	7	21	L-phenylalanine metabolic process
1,20E-07	114	1240	Regulation of cellular macromolecule biosynthetic process	1,30E-04	5	9	Cinnamic acid biosynthetic process
1,20E-07	112	1186	Regulation of RNA biosynthetic process	1,30E-04	5	9	Cinnamic acid metabolic process
1,20E-07	116	1271	Regulation of biosynthetic process	1,50E-04	8	33	Cellular amino acid catabolic process
5,50E-07	269	3771	Cellular macromolecule metabolic process	1,70E-04	29	408	Small molecule biosynthetic process

8,40E-07	114	1291	Nucleic acid-templated transcription	3,60E-04	116	2876	Biosynthetic process
8,50E-07	114	1295	RNA biosynthetic process	3,60E-04	10	63	Aromatic amino acid family metabolic process
8,50E-07	113	1279	Transcription, DNA-templated	3,60E-04	48	904	Small molecule metabolic process
1,60E-06	114	1312	Regulation of gene expression	3,60E-04	8	39	Secondary metabolite biosynthetic process
4,20E-06	121	1445	Regulation of cellular metabolic process	6,60E-04	10	69	Small molecule catabolic process
4,80E-06	117	1389	Regulation of nitrogen compound metabolic process	8,50E-04	5	14	Benzene-containing compound metabolic process
5,00E-06	118	1407	Regulation of primary metabolic process	8,50E-04	49	968	Organonitrogen compound biosynthetic process
1,10E-05	155	2013	Regulation of cellular process	1,00E-03	109	2752	Cellular biosynthetic process
2,40E-05	119	1470	Regulation of macromolecule metabolic process	1,20E-03	110	2796	Organic substance biosynthetic process
2,40E-05	156	2060	Macromolecule biosynthetic process	1,20E-03	29	471	Peptide metabolic process
2,70E-05	128	1617	Aromatic compound biosynthetic process	1,30E-03	8	49	Organic acid catabolic process
3,10E-05	123	1543	Regulation of metabolic process	1,30E-03	8	49	Carboxylic acid catabolic process
3,40E-05	153	2028	Cellular macromolecule biosynthetic process	1,30E-03	6	25	Phenylpropanoid biosynthetic process
4,60E-05	164	2223	Regulation of biological process	1,40E-03	31	529	Cellular amide metabolic process
4,60E-05	131	1689	Organic cyclic compound biosynthetic process	1,50E-03	9	65	Secondary metabolic process

4,60E-05	37	307	Response to oxygen-containing compound	1,50E-03	15	169	Alpha-amino acid metabolic process
4,60E-05	77	856	Protein phosphorylation	2,20E-03	116	3055	Organonitrogen compound metabolic process
4,70E-05	15	69	Response to fungus	2,30E-03	27	448	Translation
4,90E-05	12	45	Response to wounding	2,70E-03	6	30	Xyloglucan metabolic process
5,70E-05	7	14	Benzene-containing compound metabolic process	2,70E-03	27	454	Peptide biosynthetic process

**Molecular function**							
**24 hours**				**48 hours**			
**Enrichment FDR**	**Genes in list**	**Total genes**	**Functional Category**	**Enrichment FDR**	**Genes in list**	**Total genes**	**Functional Category**
3,80E-17	75	451	DNA-binding transcription factor activity	3,20E-06	8	19	Ammonia-lyase activity
3,80E-17	79	496	Transcription regulator activity	2,80E-05	9	35	Carbon-nitrogen lyase activity
5,80E-09	122	1295	DNA binding	3,60E-05	28	356	Structural molecule activity
1,80E-06	31	197	Calcium ion binding	4,00E-05	25	301	Structural constituent of ribosome
8,50E-06	37	281	Sequence-specific DNA binding	6,50E-05	5	9	Phenylalanine ammonia-lyase activity
6,90E-05	75	828	Protein kinase activity	3,60E-04	20	242	Lyase activity
7,60E-05	8	19	Ammonia-lyase activity	9,60E-04	6	24	Cysteine-type endopeptidase inhibitor activity
7,80E-05	12	46	Calmodulin binding	2,10E-03	6	28	Xyloglucan:xyloglucosyl transferase activity
8,80E-05	85	992	Kinase activity	2,80E-02	9	97	Magnesium ion binding
2,60E-04	79	932	Phosphotransferase activity, alcohol group as acceptor	2,80E-02	6	46	Calmodulin binding
4,00E-04	297	4633	Organic cyclic compound binding	3,00E-02	6	49	Endopeptidase inhibitor activity

4,00E-04	297	4632	Heterocyclic compound binding
4,50E-04	158	2230	Nucleic acid binding
5,80E-04	120	1614	Catalytic activity, acting on a protein
6,10E-04	5	9	Phenylalanine ammonia-lyase activity
3,20E-03	87	1147	Transferase activity, transferring phosphorus-containing groups
4,70E-03	8	35	Carbon-nitrogen lyase activity
5,80E-03	7	28	Xyloglucan:xyloglucosyl transferase activity
6,30E-03	157	2356	Transferase activity
1,30E-02	13	94	Glucosyltransferase activity
2,10E-02	27	285	Protein serine/threonine kinase activity
2,60E-02	10	68	Aspartic-type endopeptidase activity
2,60E-02	10	68	Aspartic-type peptidase activity
3,60E-02	14	120	Ubiquitin-protein transferase activity
3,70E-02	9	61	Protein serine/threonine phosphatase activity
4,50E-02	14	124	Ubiquitin-like protein transferase activity
3,00E-02	6	49	Peptidase inhibitor activity
3,00E-02	6	49	Endopeptidase regulator activity
3,10E-02	6	50	Peptidase regulator activity

Table S4. Metabolite amounts (means ± SE) expressed as relative abundance based on the relative response compared to internal standard adonitol, 24 h post application of *Bacillus subtilis* MBI600 (*Bs* MBI600).

**Substance**	**Control**	***Bs* MBI600**
	**Water-soluble sugars**	
4-Ketoglucose	0.078 ± 0.015	0.095 ± 0.0001
Ribofuranose	0.159 ± 0.039	0.102 ± 0.0001
Fructose	0.626 ± 0.014	0.723 ± 0.062
Sorbose	0.560 ± 0.045	0.662 ± 0.049
Glucose	2.922 ± 0.470	3.538 ± 0.347
Mannose	0.456 ± 0.053	0.600 ± 0.053
Talopyranose	0.126 ± 0.016	0.163 ± 0.0001
Mannopyranoside	0.096 ± 0.008	0.127 ± 0.0002
Galactopyranoside	0.227 ± 0.077	0.233 ± 0.055
D-Allose	nd[a]	0.190 ± 0.027
Sucrose	0.194 ± 0.067	0.172 ± 0.013
	**Sugar alcohols**	
Mannitol	0.125 ± 0.0003	0.114 ± 0.009
Glycerol	1.236 ± 0.086	0.997 ± 0.108
Myo inositol	1.889 ± 0.063	2.229 ± 0.347
	**Other organic substances**	
Aucubin	1.735 ± 0.258	1.739 ± 0.519
Gluconic acid, γ-lactone	nd	0.171 ± 0.044
Phosphoric acid	2.160 ± 0.103	1.826 ± 0.175

Urea	0.460 ± 0.155	nd
**Organic acids**		
Malic acid	0.923 ± 0.128	4.591 ± 0.189*[b]
Threonic acid	0.080 ± 0.0002	0.097 ± 0.006
Erythronic acid	0.249 ± 0.018	0.350 ± 0.022*
Glyceric acid	2.068 ± 0.569	5.721 ± 0.529*
Octadecatrienoic acid	0.088 ± 0.015	0.112 ± 0.002
Arabinonic acid	0.088 ± 0.0007	0.103 ± 0.0001*
Butanoic acid	0.170 ± 0.006	0.229 ± 0.005*
Ribonic acid	0.239 ± 0.097	0.088 ± 0.010
Gluconic acid	0.252 ± 0.177	0.109 ± 0.005
Citric acid	0.302 ± 0.040	0.479 ± 0.09[illegible]
Hexadecanoic acid	0.098 ± 0.023	nd
Linoleic acid	0.116 ± 0.018	0.117 ± 0.020
**Amino acids**		
Alanine	0.653 ± 0.133	1.134 ± 0.120*
Valine	0.247 ± 0.069	0.208 ± 0.018
Citrulline	0.131 ± 0.024	nd
Norleucine	0.221 ± 0.027	0.166 ± 0.023
Glycine	0.140 ± 0.029	0.125 ± 0.015
Oxoproline	0.126 ± 0.023	0.174 ± 0.025
Serine	0.195 ± 0.052	0.133 ± 0.0001
Proline	0.989 ± 0.215	0.586 ± 0.139
Threonine	0.407 ± 0.103	0.379 ± 0.048
Glutamic acid	1.425 ± 0.336	1.204 ± 0.121
Aspartic acid	0.468 ± 0.088	0.573 ± 0.051

Gamma-aminobutyric acid (GABA)	1.018 ± 0.146	0.853 ± 0.065

[a]nd stands for not detected

[b]Asterisks indicate values that differ significantly at each time point (pairwise t-test, $P \leq 0.05$)

Table S5. Metabolite amounts (means ± SE) expressed as relative abundance based on the relative response compared to internal standard adonitol 48 h post application of *Bacillus subtilis* MBI600 (*Bs* MBI600).

**Substance**	**Control**	***Bs* MBI600**
**Water-soluble sugars**		
4-Ketoglucose	0.087 ± 0.013	0.084 ± 0.012
Ribofuranose	0.117 ± 0.034	0.065 ± 0.011
Fructose	0.629 ± 0.121	0.326 ± 0.020*[b]
Sorbose	0.698 ± 0.050	0.389 ± 0.079*
Galactose	nd[a]	0.036 ± 0.010
Glucose	2.973 ± 0.569	1.610 ± 0.104
Mannose	0.543 ± 0.066	0.310 ± 0.023*
Talopyranose	0.188 ± 0.024	0.097 ± 0.009*
Xylopyranose	nd	0.150 ± 0.066
Mannopyranoside	0.146 ± 0.027	0.313 ± 0.205
Galactopyranoside	0.302 ± 0.026	0.142 ± 0.019
Ribose	0.117 ± 0.020	0.075 ± 0.011
D-Allose	0.115 ± 0.082	0.107 ± 0.0001
Allopyranose	0.081 ± 0.010	0.045 ± 0.008*
Sucrose	0.433 ± 0.090	0.147 ± 0.042
**Sugar alcohols**		
Mannitol	0.228 ± 0.017	0.110 ± 0.019*
Glycerol	1.143 ± 0.111	1.172 ± 0.103
Myo inositol	2.740 ± 0.349	1.270 ± 0.309*
**Other organic substances**		

Aucubin	0.529 ± 0.122	0.154 ± 0.083*
Gluconic acid, γ-lactone	nd	0.085 ± 0.036
PhosphoricAcid	1.908 ± 0.199	1.959 ± 0.184
Urea	nd	0.171 ± 0.031
**Organic acid**		
Malic acid	2.627 ± 0.531	5.011 ± 0.518
Threonic acid	0.190 ± 0.072	0.074 ± 0.003*
Erythronic acid	0.276 ± 0.024	0.368 ± 0.018*
Glyceric acid	nd	0.036 ± 0.005
Octadecatrienoic acid	0.095 ± 0.006	0.082 ± 0.010
Citric acid	0.302 ± 0.040	0.479 ± 0.096
Arabinonic acid	0.066 ± 0.013	0.073 ± 0.011
Butanoic acid	0.160 ± 0.0001	0.130 ± 0.007*
Ribonic acid	0.118 ± 0.032	0.116 ± 0.010
DeoxyErythroPentonic acid	0.187 ± 0.040	0.092 ± 0.021
ButanedioicAcid	0.078 ± 0.009	0.109 ± 0.009
Glyceric acid	2.070 ± 0.583	3.132 ± 0.474
Butanoic acid	nd	0.067 ± 0.023
Gluconic acid	0.093 ± 0.012	0.048 ± 0.005
Linoleic acid	0.227 ± 0.070	0.113 ± 0.014
**Amino acids**		
Alanine	0.568 ± 0.074	0.896 ± 0.119*
Isoleucine	nd	0.078 ± 0.013
Valine	0.232 ± 0.032	0.435 ± 0.078*
Citrulline	nd	0.043 ± 0.007
Norleucine	0.203 ± 0.037	0.357 ± 0.063

Glycine	0.098 ± 0.016	0.149 ± 0.026
Oxoproline	0.334 ± 0.084	0.249 ± 0.041
Serine	0.161 ± 0.019	0.093 ± 0.019*
Proline	0.236 ± 0.045	0.330 ± 0.063
Threonine	nd	0.037 ± 0.005
Asparagine	0.113 ± 0.023	0.100 ± 0.012
Leucine	0.204 ± 0.029	0.272 ± 0.042
Glutamic acid	0.500 ± 0.055	0.652 ± 0.100
Aspartic acid	1.177 ± 0.250	1.289 ± 0.208
Gamma-aminobutyric acid (GABA)	0.522 ± 0.042	1.169 ± 0.091*

[a]nd stands for not detected

[b]Asterisks indicate values that differ significantly at each time point (pairwise t-test, $P \leq 0.05$)
