# Supplementary figures and images for: Root Transcriptional and Metabolic Dynamics Induced by the Plant Growth Promoting Rhizobacterium (PGPR) Bacillus subtilis Mbi600 on Cucumber Plants
Source: Plants (Basel). 2022 Apr 30;11(9):1218. doi: 10.3390/plants11091218 (PMC9102019; doi:10.3390/plants11091218)

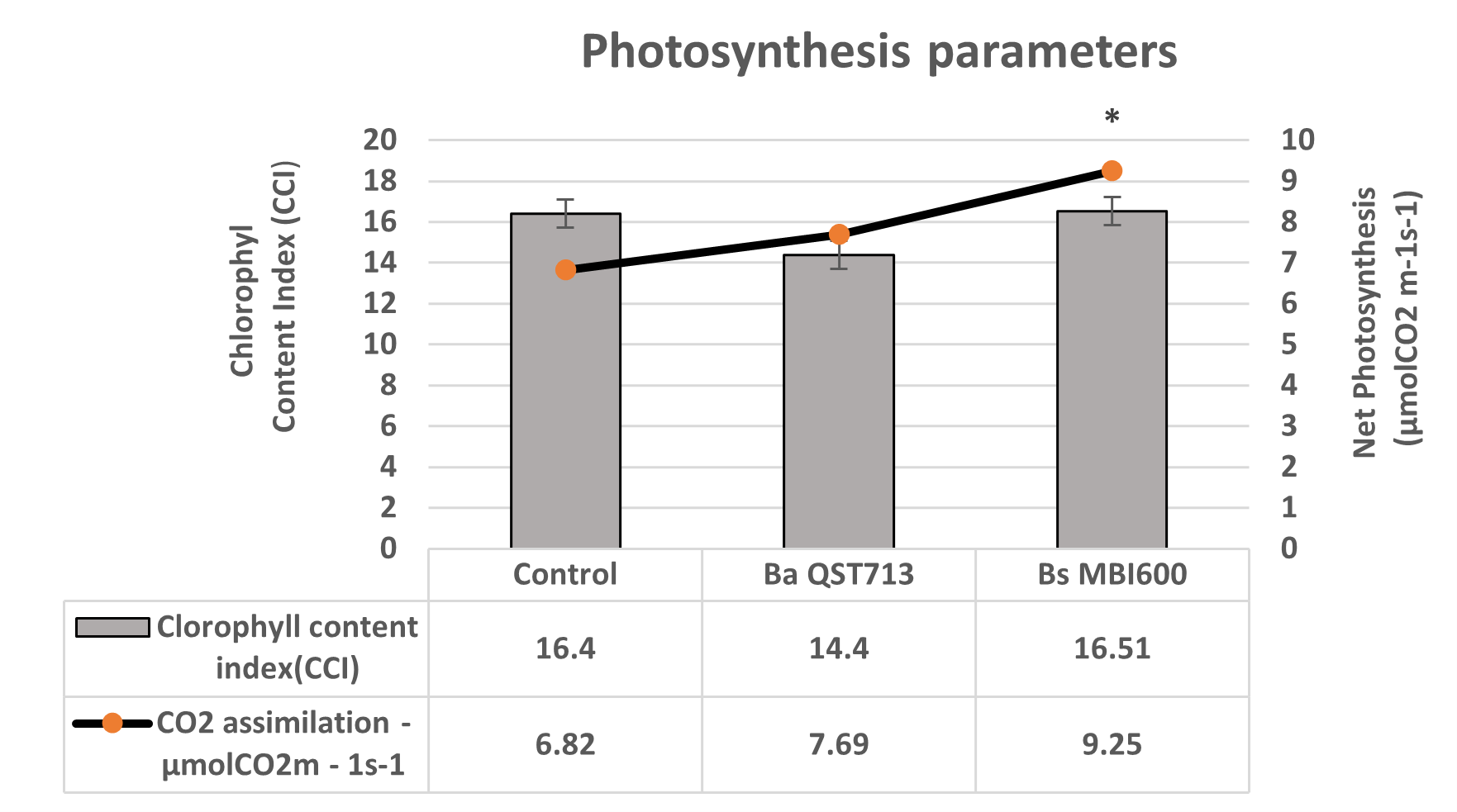

Supplement: Supplementary file 1 [file plants-11-01218-s001.zip › Figure S1.tiff]
